# A systems approach to model natural variation in reactive properties of bacterial ribosomes

**DOI:** 10.1186/1752-0509-2-62

**Published:** 2008-07-13

**Authors:** Julius H Jackson, Thomas M Schmidt, Patricia A Herring

**Affiliations:** 1 Department of Microbiology & Molecular Genetics, Michigan State University, East Lansing, MI 48824, USA; 2 Theoretical and Computational Biology Group, Department of Microbiology & Molecular Genetics, Michigan State University, East Lansing, MI 48824, USA

## Abstract

**Background:**

Natural variation in protein output from translation in bacteria and archaea may be an organism-specific property of the ribosome. This paper adopts a systems approach to model the protein output as a measure of specific ribosome reactive properties in a ribosome-mediated translation apparatus. We use the steady-state assumption to define a transition state complex for the ribosome, coupled with mRNA, tRNA, amino acids and reaction factors, as a subsystem that allows a focus on the completed translational output as a measure of specific properties of the ribosome.

**Results:**

In analogy to the steady-state reaction of an enzyme complex, we propose a steady-state translation complex for mRNA from any gene, and derive a maximum specific translation activity, *T*_*a*(max)_, as a property of the ribosomal reaction complex. *T*_*a*(max) _has units of *a*-protein output per time per *a*-specific mRNA. A related property of the ribosome, ${\tilde{T}}_{a(\max)}$, has units of *a*-protein per time per total RNA with the relationship ${\tilde{T}}_{a(\max)}$ = *ρ*_*a *_*T*_*a*(max)_, where *ρ*_*a *_represents the fraction of total RNA committed to translation output of *P*_*a *_from gene *a *message. *T*_*a*(max) _as a ribosome property is analogous to *k*_cat _for a purified enzyme, and ${\tilde{T}}_{a(\max)}$ is analogous to enzyme specific activity in a crude extract.

**Conclusion:**

Analogy to an enzyme reaction complex led us to a ribosome reaction model for measuring specific translation activity of a bacterial ribosome. We propose to use this model to design experimental tests of our hypothesis that specific translation activity is a ribosomal property that is subject to natural variation and natural selection much like *V*_max _and *K*_m _for any specific enzyme.

## Background

Measures of primary sequence variation in ribosomal RNA, ribosomal protein, and non-ribosomal enzyme protein show that the corresponding gene sequences provide a record of natural variation associated with phylogeny in bacteria and archaea [1,2]. Recent analysis of a select subset of 32 protein-coding genes, mostly associated with transcription and translation machinery, established phylogenetic relationships among the host genomes of 69 bacterial and archaeal organisms, and the basis for a molecular tree of variation calibrated to geological time [1]. The phylogenetic tree constructed in that study largely resembles trees based upon analyses of 16S rDNA sequences [3].

In addition to the phylogenetic perspective based on sequence variation in ribosomal RNA and ribosomal protein encoding genes, we suggest that this sequence variation underlies differences in the performance of the translational machinery. Modeling variation in the kinetics of translation, the primary energy-requiring process of microbes, should lead to an enhanced ecological perspective of microbial life, much like enzyme kinetics has provided the basis for physiological models of metabolism.

Variation in kinetic properties of enzymes has been associated with phylogenetic variation in closely related bacteria but data are less readily available for the variation in enzyme families. In some few studies, *K*_*m *_and *V*_max _have been used as functional parameters of enzymes to infer neutral or adaptive properties associated with allelic variation in natural populations [4,5]. Hereafter, we refer to the kinetic parameters of enzymes as specific reactive properties. These studies suggest that we should expect phylogenetic variation in measures of specific reactive properties, e.g. *K*_*m*_, *V*_max_, and *k*_cat_, for a family of functionally conserved enzymes. Though a recent, systematic study of the natural variation in specific reactive properties of enzyme families does not appear to be available, a brief survey of literature supports the expectation for natural variation of *K*_*m*_, *V*_max _in an enzyme family. For example, the published values for *K*_m _of biosynthetic threonine deaminase (EC 4.2.1.16) vary from 0.25 mM in *Thiobacillus acidophilus *and 3.2 mM in *Proteus morganii*, to 8.0 mM in *Escherichia coli*, 13.9 mM in *Geobacillus stearothermophilus*, and 14.0 mM in *Thermus *sp. [6-10]. Similarly, the *V*_max _value for purified threonine deaminase varies over a range from 2.1 μmol/min/mg in *Pseudomonas putida *to 210 μmol/min/mg in *E. coli *and even higher in other organisms. Overall, the reactive properties of the biosynthetic threonine deaminase (4.2.1.16) family of enzymes appear to reflect a natural variation over an approximate range greater than two orders of magnitude. Simply stated, enzymes and enzyme complexes that evolve to carry out the same reactions in a set of organisms, should be expected to catalyze these reactions with sometimes substantial differences in steady-state rates and reaction efficiencies, owing to natural variation in sequence associated with reaction properties. Variation in reaction properties may confer a fitness advantage on organisms and be fixed rapidly in populations, or be selectively neutral and accumulate in populations as a result of genetic drift.

In analogy to the natural variation of conserved reactive properties of proteins, ribosomal RNA sequences undergo natural variation that forms the basis for phylogenetic grouping of all organisms and reflects the physiological adaptations associated with the sequence variation [9]. Regions of rRNA that are most highly conserved are speculated to be associated with the ribosomal reaction properties moreso than the regions which are more free to vary. Ribosomal proteins also reflect sufficiently conserved variation that allows for their use in phylogenetic grouping [1]. Since phylogenetic variation in enzyme protein sequence is associated with variation in measurable, reactive properties of the enzymes, the suggestion seems reasonable that measurable, reactive properties of ribosomes are associated with regions of rRNA and ribosomal protein sequences that undergo natural variation. Thus, reactive properties of ribosomes should also be expected to vary in ways similar to the natural variation observed for enzymes [11].

A small, phylogenetically varied subset of bacteria, grouped by fast or slow growth rates in response to nutrient availability, demonstrated variation in a measurable property of ribosomal activity termed translational power by Dethlefsen and Schmidt [12]. This translational power displayed natural variation over approximately a five-fold range [11]. By inference from those studies, we postulate that specific reactive properties of ribosomes are measurably constant for any specific bacterial phylotype or species in a constant environment, and that each reactive property is subject to natural variation under selective constraints to maintain function.

In the present study we consider the ribosome and associated translational machinery as a co-evolving complex of functionally conserved molecules with measurably varied reactive properties in distinct bacterial populations. Consequently, we expect to find that ribosomes from different bacteria vary in their steady-state output of proteins as a result of selective pressures that have fine-tuned the translational machinery for optimal activity in a given environment. In analogy to a multi-enzyme complex, we model ribosomal catalyzed translation activity as a steady-state process involving the entire translational complex as a transition-state intermediate such that the measurably important output is a functional protein. The translational apparatus complex is, then, a subsystem of the organism and produces the mass ratios of proteins required for maintenance and growth. Treatment of the translational complex as a subsystem operating in a steady-state is the key factor that makes measurement of protein output sufficient to characterize subsystem properties associated with a ribosome. Our model suggests that variation in the reactive properties of ribosomes can be detected by measuring the steady-state output of specific, functional proteins. This model substitutes simpler, systems measures to replace the more complex, step-wise, mechanistic measures used elsewhere, [e.g. [13,14]]. Details of the design strategy to develop this measure follow.

## Methods

### Strategy to measure reactive properties of ribosomes

In order to measure specific reactive properties of ribosomes, the design of a model of the translational apparatus as a bacterial subsystem must take into account all factors that can influence the rate of protein output that establishes gene expression levels and the mass ratio of proteins for steady-state growth. When all such factors can be held constant, then measurement of translational output records a ribosome-specific property. Under any steady state growth condition the properties of transcription and translation machinery are constant. A few definitions, here, are useful to build a model. For this model we define expression level as the steady state mass ratio of protein, *P*_*a*_, from a specific gene compared to the total cell protein, *P*, i.e. *P*_a_/*P*.

The instantaneous mass ratio or expression level of a protein from any gene in a bacterium under steady state growth conditions is determined by probabilities affecting the following steps: 1, transcription initiation (promoter strength, promoter configuration, transcription factors; and metabolites); 2, transcription termination; 3, mRNA stability; 4, ribosome binding site (e.g. Shine-Dalgarno sequence) affinity for ribosome; 5, ribosome loading at the Shine-Dalgarno (SD) or other sequences; 6, relative codon frequency pattern, i.e. codon usage; 6, translation initiation (start codon; initiation factors); 7, protein elongation (GTP, aminoacylated tRNA, elongation factors), and termination; 8, frequency of polypeptide completion; 9, protein folding; 10, protein stability; and 11, metabolite balance regulating enzyme activity and synthesis [15-18].

**Figure 1:**
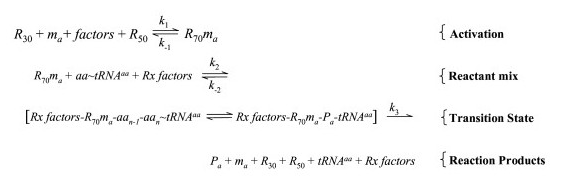
**Ribosome reaction model, analogy to an enzyme complex.** Abbreviations: *R*_30_, 30s ribosomal subunit; *m*_*a*_, mRNA from gene *a*; *R*_50_, 50s ribosomal subunit; *aa*, amino acid; *tRNA*^*aa*^, tRNA isoaccepting species for amino acid *aa*; *aa*~*tRNA*^*aa*^, aminoacylated tRNA; *Rx*, reaction; and *P*_*a*_, protein from translation of mRNA from gene *a*.

For this study, transcription processes in steps 1 and 2 are held constant, and steps 3–11 form the basis to model the steady-state output activity of the translation apparatus. Fig. 1 and the accompanying description refer to these steps 3–11.

### Rationale for the model

In the exponential state of bacterial growth in a closed system, the rate of production of cell mass, *dx/dt*, is proportional to the initial cell mass, *x*, with the proportionality represented by the instantaneous growth rate constant, *k*, as

(1) *dx/dt *= *kx*.

In this steady state, the rate of production of any component of cell mass is commonly and reasonably assumed to be a constant fraction of the total cell mass, and *k *would be the expected proportionality constant for production of any component of the cell. This is a reasonable assumption and subject to a simple proof that need not be shown here. From this inference, as suggested by Neidhardt et al. [19], *k *defines the steady-state rate of protein production in Eq. (2).

(2) $$k=\frac{1}{x}\frac{dx}{dt}=\frac{1}{P}\frac{dP}{dt}$$

In the steady state translation output of a genome represented by Eq. (2), the protein output per unit total RNA is

(3) $$T=\frac{kP}{R},$$

where *T *= the protein translation output of a genome, measured in units of total protein, *P*, per unit total RNA, *R*, in a bacterium in steady state growth with instantaneous growth rate, *k *[12,20]. This relationship approximates a property of average translation activity per unit total RNA for an organism in a specific state of growth. We might also refer to *T *as the translation completion rate in recognition that not all starts of polypeptide synthesis reach completion [18]. Considering the protein product from a single gene as a regulated, fractional component of cell mass, Eq. (2) implies that the change in the mass of protein, *P*_*a*_, from any gene *a *in genome *A*, i.e. *a *∈ *A*, is

(4) *dP*_*a*_/*dt *= *kP*_*a*_.

It follows that a translation output *T*_*a*_, from any gene *a *per unit total RNA results from translation activity by the subset of the ribosomes translating the message from gene *a*. Ribosomal RNA constitutes approximately 85% of total RNA in bacteria [21], so total RNA is mostly ribosomal. Let *ρ*_*a *_represent the fraction of total RNA committed to translation output of *P*_*a *_from gene *a *message, then the amount of *R *committed to producing *P*_*a *_is *ρ*_*a*_*R*. Thus *ρ*_*a*_*R *approximates the fraction of ribosomes translating the mRNA from gene a *m*_*a*_. Since Eq. (3) defines the general relationship of the overall translational output to total RNA in a growing organism or culture, i.e. the translational power, we propose Eq. (5) as a special case of Eq. (3) to represent the specific translational output from any one specific gene, *a*.

(5) $$T_{a}=\frac{kP_{a}}{\rho_{a}R}$$

## Results

### Ribosome analogy to an enzyme complex

Through simplifying assumptions, treat the 70S ribosome as a multi-subunit enzyme that catalyzes reactions to polymerize amino acids to make a functional protein product, *P*_*a*_. A summary representation of this process is diagrammed as a chemical reaction sequence (Fig. 1). For any gene, treat the initiation process and assembly of the 70S ribosome as analogous to activation of the ribosome as an enzyme. The formation of the initiation complex involves binding of 30S ribosome (*R*_30_) with mRNA from gene *a*, (*m*_*a*_), at a Shine-Dalgarno (SD) or other ribosome binding site; f-met~tRNA; the initiation factors IF1, IF2 and IF3; GTP; and subsequent assembly with 50S ribosome (*R*_50_) to form the activated ribosome, *R*_70_*m*_*a*_. In this analogy, reaction cofactors are not rate limiting, *m*_*a *_is an activator, and aminoacylated tRNAs (aa~tRNA^aa^) are substrate molecules. The amino acid polymerization reactions that follow utilize the pools of aa~tRNA^aa ^to produce the polypeptides for the functional *P*_*a *_protein. Treat all steps of polypeptide chain elongation reactions as a single transition state intermediate. The overall reaction product is *P*_*a*_, formed in a step involving release from the ribosome-mRNA complex and assembly of the active protein. We consider the last step as rate-determining. Measurement of the rate of increase of *P*_*a *_reactivity is the measure of the rate of production of functional proteins from translation of gene *a *message.

Continuing the analogy, the active ribosome complex, *R*_70_*m*_*a*_, would be expected to vary the rate of polypeptide output in response to the variables of input, especially the pool of aa~tRNA^aa^, and the codon composition of the gene transcript, *m*_*a*_. The rate of the translation output would be expected to vary as a function of the degree of match of the aa~tRNA^aa ^composition and abundance with the relative codon frequency of the transcript. We posit that a major source of the variation of specific translation activity is the degree of mismatch between the relative codon frequency and the tRNA abundance and composition. The greater the degree of mismatch, the higher the frequency of codon-specific pauses and the greater is the probability of premature termination of polypeptide synthesis, i.e. a higher drop-off frequency. Thus, within a genome, *A*, the translation activity of its ribosomes would be expected to vary over a definable range up to a maximum translation activity that is a property of the ribosome.

The rate of protein output in this Ribosome Reaction Model is a function of the catalytic capacity of the activated ribosome complex acting upon the pool of aa~tRNA^aa ^as shown by Eq. (6).

(6) *dP*_*a*_*/dt *= *k*_3_[R_70_*m*_*a*_]

We can see that the rate of production of *P*_*a *_is the initial reaction velocity of output from the gene *a *message, thus

(7) *dP*_*a*_/*dt *= *v*_*a*_.

From Eqs. (6–7) we see that

(8) *k*_3 _= *v*_*a*_/[*R*_70_*m*_*a*_].

Eqs. (4–6) imply that $T_{a}=\frac{k_{3}[R_{70}m_{a}]}{\rho_{a}R}$, and from our definition of *ρ*_*a*_*R *as the amount of *R *involved with translation of mRNA from gene *a*, we infer that *ρ*_*a*_*R *≈ [*R*_70_*m*_*a*_] and thus *k*_3 _≈ *T*_*a*_, the specific translation activity. The units for *k*_3 _are units of *P*_*a *_formed per unit time per unit *R *involved in translating message *m*_*a *_from gene *a*. These units are the same as the units for *T*_*a*_, the specific translation activity in Eq. (5). Under conditions where [*R*_70_*m*_*a*_] is maximum, all mRNA from gene *a *is loaded onto ribosomes, and the maximum output rate is achieved, then *v*_*a *_becomes *V*_*a*(max)_. For conditions producing *V*_*a*(max)_, *k*_3 _becomes analogous to *k*_cat _for an enzyme reaction, as in Eq. (9).

(9) *k*_cat _= *V*_*a*(max)_/[*R*_70_*m*_*a*_].

Activated ribosomes within an organism will therefore be expected to display a property of maximum specific translation activity, *T*_*a*(max)_, under optimal conditions of reactant availability. By inference, it may be possible to measure the catalytic efficiency for use of any specific aa~tRNA^aa ^in translation of *m*_*a *_by measure of *k*_cat_/*K*_m_, i.e. *T*_*a*(max)_/*K*_m_. We might expect, then, that the translation properties of ribosomes in a *R*_70_*m*_*a *_complex are constant, are organism specific, and therefore are genome-specific. *k*_cat _for the complex would be expected to vary according to *m*_*a *_properties that influence the expression level.

### Single gene translation activity, factors and determinants

Modifying Eq. (5) to introduce a term for the expression level of gene *a*, define the specific translation activity for gene *a *as

(10) $$T_{a}=\frac{kP_{a}}{\rho_{a}R}=\frac{kP\lambda_{a}}{\rho_{a}R}$$

where *λ*_*a *_= *P*_*a*_/*P *is the expression level. The *λ*_*a *_term shows the fractional contribution of functional protein from any gene, *a*, to the total protein content of the organism in any steady state of a growth cycle. Thus, *λ*_*a *_is the mass ratio of protein *P*_*a *_compared to the total cellular protein. The *dP*_*a*_/*dt *varies among the genes of a genome such that a gene or gene set that is highly expressed has a higher production rate for *P*_*a *_than genes expressed less frequently. Therefore, multiple expression states must be expected to exist according to the functional role of a gene or set of genes in the life cycle of an organism. The consequence of these multiple expression states is that the steady state translation of transcripts from any low expression gene contributes fewer proteins than a high expression gene to the total cellular protein.

The half-life of proteins that comprise *P *would be expected to vary over some range and that variation will constitute some frequency distribution. Considering the variation in stability of any protein *P*_*a*_, a more accurate representation of *λ*_*a *_= *P*_*a*_/*P *may be

(11) $$\lambda_{a}=\frac{P_{a}}{\sum P_{a}}.$$

In theory, the measurable total protein mass, *P*, will be experimentally indistinguishable from ∑*P*_*a *_even if the *t*_1/2 _of one or several *P*_*a *_is extremely short. Therefore, we expect that *P *≈ ∑*P*_*a*_. However, in the case of an extremely short half-life for any *P*_*a*_, the expression of *P*_*a *_would appear to be low and lead to an erroneous conclusion that the mass ratio *λ*_*a *_is low. Though a condition of high instability of *P*_*a *_may be rare, calculation of the initial mass ratio from Eq. (11) can be accomplished by substituting the initial protein output *P*_*a*' _for *P*_*a*_,

(12) $$P_{a'}=P_{a}e^{c_{a}t},$$

where *c*_*a *_is a protein stability constant, and *t *is the time increment between completion of protein synthesis and sampling. These relationships derive from treating the decay of the functional protein *P*_*a *_as *P*_*a *_= *P*_*a*' _exp-(*c*_*a*_*t*). Note that initially, i.e. when *t *= 0, the measured value and initial value for *P*_*a *_are equal, and when *c*_*a *_is small, i.e. stable protein, *P*_*a *_≈ *P*_*a*'_. Thus *P*_*a*' _is an estimate of the original protein mass when *t*, *c*_*a*_, and *P*_*a *_are known.

Since not all translation starts, $P_{a_{s}}$, reach completion for a gene message owing to premature termination, *ψ*_*a *_in Eq. (13) represents a translation completion efficiency for each gene.

(13) $$\psi_{a}=\frac{P_{a}}{P_{a_{s}}}$$

Then *δ*, a so-called "drop-off frequency" sometimes used to describe translation efficiency, may be defined as

(14) $$\delta_{a}=1-\psi_{a}=1-\frac{P_{a}}{P_{s}}.$$

For unstable *P*_*a*_, i.e. when the protein stability constant *c*_*a *_is large, then it would be appropriate to substitute *P*_*a*' _for *P*_*a *_from Eq. (13) and to introduce an initial Pas' to substitute for $P_{a_{s}}$ to get

(15) ψa'=Pa′Pas'

where

(16) Pas'=Pasecat.

The rationale for Eq. (16) is analogous to that for Eq. (12), and Pas' is an estimate of the original protein mass from completion of all translation starts when *t*, *c*_*a*_, and $P_{a_{s}}$ are known. Generally, it would be appropriate to use the efficiency factor of Eq.(13) and reserve Eq. (15) for probably rare instances of a highly unstable *P*_*a*_.

We may now also define specific translation activity for a single gene *a *as

(17) $$T_{a}=\frac{kP_{a_{s}}\psi_{a}}{\rho_{a}R},$$

and when considering the instability of proteins from any gene *a*,

(18) Ta=kPas'ψa'ρaR.

### Ribosome loading and transcript stability

We will next treat, specifically, the initiation step, also called the ribosome loading step. Thus far we have considered factors affecting the specific translation activity, *T*_*a*_. The overall translation rate, however, also depends upon the frequency of ribosome loading onto mRNA, the stability of that mRNA, and the probability and frequency of the gene transcription. The first step of the reaction sequence (Fig. 1) represents ribosomal loading onto transcript *m*_*a *_from gene *a *∈ *A*, described by rate equation Eq. (19).

(19) $$\frac{d[R_{70}m_{a}]}{dt}=k_{R}[R_{30}][m_{a}][R_{50}]-k_{-1}[R_{70}m_{a}]$$

In Eq. (19), *k*_*R *_is a third-order rate constant with units of (*t*^-1^)(conc^-1^)(conc^-1^). The conditions of growth result in [*m*_*a*_] << [*R*_30_] and [*m*_*a*_] << [*R*_50_]. These conditions suggest that the association reaction may display first-order behavior. Let *k*_+1 _be a pseudo first-order rate constant, where *k*_+1 _= *k*_*R *_[*R*_30_] [*R*_50_], and substitute into Eq. (19) to obtain

(19a) $$\frac{d[R_{70}m_{a}]}{dt}=k_{+1}[m_{a}]-k_{-1}[R_{70}m_{a}].$$

We see then that Eq. (19a) shows the expected pseudo first-order behavior of *m*_*a *_binding to ribosomes. At any steady state of growth, ribosomal activation can be represented by an association constant, *K*_*a*_, for the message *m*_*a *_from gene *a*, as

(20) $$K_{a}=\frac{k_{+1}}{k_{-1}}=\frac{\left[R_{70}m_{a}\right]}{\left[m_{a}][R_{30}][R_{70}\right]}.$$

This association constant can be thought of as a loading factor that is a ratio of bound to free mRNA from gene *a*, and can be related to the probability of message binding to ribosome. The amount of *m*_*a *_undergoing translation compared to the total *m*_*a *_is the relative frequency of loading and approximates the probability of ribosomal loading, i.e.

(20a) $$\pi_{a}\approx f_{1}=\frac{\left[R_{70}m_{a}\right]}{\left[m_{a}]+[R_{70}m_{a}\right]},$$

where *f*_*l *_is the relative loading frequency and *π*_*a *_is the probability of message from gene *a *binding to ribosomes for translation. The specific translation rate, *T*_*a*_, weighted by the fraction of total message loaded, *π*_*a*_([*m*_*a*_] + [*R*_70_*m*_*a*_]) = *π*_*a*_*m*_*a*(total)_, gives an overall expectation for translation rate of gene a message to make the functional protein, *P*_*a *_(Eq. (21)).

(21) *T*_*rate *_= *π*_*a*_*m*_*a*(total)_*T*_*a*_

Factors and growth conditions that affect transcription initiation are too numerous and variable to treat in the context of overall translation rate. Therefore, the last step we consider in this treatment of the overall specific translation rate, *T*_rate_, is transcript stability. The higher the stability of *m*_*a*_, the greater is the output of *P*_*a *_per time, and conversely, the lower the stability of *m*_*a*_, the lower the overall translation rate. Analogous to treatment of protein stability in Eq. (12), *m*_*a *_represents the amount of mRNA from gene *a *that is available to bind a ribosome. It is easy to see that instability of *m*_*a *_reduces the amount of transcript loaded for translation and reduces the overall translation rate. If the stability of mRNA, i.e. half-life, for gene *a *is known, it should be possible to use Eq. (22) to estimate the actual *m*_*a *_available for loading,

(22) $$m_{a}=m_{a'}e^{-\omega_{a}t},$$

where *ω*_*a *_is a constant of mRNA stability for gene *a*, and *m*_*a*' _is the initial amount of message.

### Maximum translation rate

Ideal conditions that include optimal mRNA stability and protein stability would be expected to maximize production of proteins from a single, protein-coding gene. Every translation start results in a completed protein under optimal conditions when *ψ*_*a *_= 1.0. Thus, an expected maximum, steady state specific translation activity by any ribosome to make any *P*_*a *_may be defined as a maximum specific translation activity

(23) $$T_{a(\max)}=\frac{kP_{a_{s}}}{\rho_{a}R}.$$

To approximate a *T*_max _for any gene *a *under non-ideal laboratory or natural conditions we may use Eq. (18) to correct for protein instabilities and translation efficiency to establish that

(24) Tmax⁡=lim⁡ψa'→1.0Ta=lim⁡ψa'→1.0(kPas'ψa'ρaR)=kPas'ρaR

is the maximum speed at which ribosomes can translate any gene in one organism at a steady state of growth. Such optimal conditions imply existence of a *K*_*a*'_, a maximum efficiency of ribosome binding to mRNA such that

(25) $$K_{a'}=\frac{\left[R_{70}m_{a'}\right]}{\left[m_{a'}][R_{30}][R_{50}\right]},$$

where $m_{a'}=m_{a}e^{\omega_{a}t}$ and this also implies a corresponding *π*_*a*' _for the *K*_*a*'_. It follows, then, from Eqs. (24) and (25) that a maximum overall translation output rate is given by

(26) *T*_rate (max) _= *π*_*a*' _*m*_*a*(total) _*T*_max_.

It is now possible to account for the multiple effect(s) of growth conditions on *T*_*a*_, including factors that affect translation efficiency, e.g. the drop-off rate and completion rate.

As defined in Eq. (24), the maximum specific translation output activity for a ribosome, *T*_max_, defines a genome-specific property as suggested by Dethlefsen and Schmidt [11]. As a general property, *T*_max _defines a capacity for production of protein, and that capacity will variably express in relation to the transcript composition from a particular gene. A convenient and practical way to look at specific translation output capacity for a single gene tied to the steady state growth rate of the organism is to combine the factors that cause variability from gene to gene within a genome into the expression for maximum specific translation output activity per unit total RNA in translation of *m*_*a*_. From Eq. (23) for expression of protein from a specific gene, *a *in genome *A*, we can see that

(27) $$T_{a(\max)}=\frac{kP_{a_{s}}}{\rho_{a}R}=\frac{kP\lambda_{a}}{\rho_{a}R\psi_{a}},$$

and in this form it is not necessary to know or measure protein starts from gene *a*, i.e. $P_{a_{s}}$, as long as the expression level, *λ*_*a*_, and the translation completion efficiency, *ψ*_*a*_, are known. In the section on Discussion and Conclusions we suggest a way to estimate a value for *ψ*_*a*_. When *ρ*_*a *_is known, i.e. the approximate fraction of ribosomes translating *m*_*a*_, the value of *T*_*a*(max) _will be highest because it is a measure of the protein expression per unit of total RNA translating *m*_*a*_. When *ρ*_*a *_is not known, a fractional approximation of *T*_*a*(max) _is

(28) $${\tilde{T}}_{a(\max)}=\frac{kP\lambda_{a}}{R\psi_{a}}$$

which has units of units *a*-protein per time per unit total RNA. It is clear that ${\tilde{T}}_{a(\max)}$ <*T*_*a*(max) _because the approximation of Eq. (28) compares the gene *a*-protein output rate to the total translational capacity as opposed to the fraction of capacity, *ρ*_*a*_, committed to *m*_*a *_translation in Eq. (27). Thus, *ρ*_*a *_relates ${\tilde{T}}_{a(\max)}$ to *T*_*a*(max)_, by ${\tilde{T}}_{a(\max)}$ = *ρ*_*a *_*T*_*a*(max)_.

The maximum specific translation output rate of functional, gene *a *protein can be measured in multiple organisms as a means to compare this ribosome-specific property among the set of organisms by using Eq. (27). As long as the functional proteins measured derive from an orthologous set of genes, the actual measurement of *T*_*a*(max) _should reveal the variation in the specific translation rate property of ribosomes from one organism with respect to that property in every other organism. Recognizing that the approximation ${\tilde{T}}_{a(\max)}$ is always less than but fractionally related to *T*_*a*(max) _by *ρ*_*a *_to a specific gene *a*, we suggest that this approximation will provide a basis to detect, quantify and compare variation in the specific translation rate property of a ribosome. We suggest that ${\tilde{T}}_{a(\max)}$ measures the specific translation activity of ribosomes analogous to enzyme specific activity measurements in crude extracts, e.g. *V*_max_/*P*, and that *T*_*a*(max) _is analogous to the *k*_cat _of a purified enzyme, e.g. *V*_max_/*P*_*a*_. We propose that Eq. (27) is an idealized mathematical model of the organism-specific translation output rate as a specific, functional property of the ribosome and that Eq. (28) is a readily measurable, fractional approximation.

## Discussion and conclusion

Analogy to the reaction of an enzyme complex formed the basis to derive Eq. (27) as a simple model for specific translation activity as a specific reaction property of a ribosome. The measure of *T*_*a*(max) _is made by the measure of *kP*/*ρ*_*a*_*R *and *λ*_a_/*ψ*_*a *_for the species-specific reaction property of a ribosome. Factors that make the measure species-specific are the expression level of the protein measured, captured in *λ*_*a *_as the relative ratio of *P*_*a *_to the total protein *P,* and the translation completion efficiency, *ψ*_*a*_. This model of translation activity of a ribosome provides a way to compare ribosome function in one bacterium to another, in bacteria that differ in their growth responses to changing environments or growth conditions. A key postulate in deriving this model is the steady-state assumption that for the time interval of constant growth conditions, the level of biosynthetic intermediates is constant and sufficient to support growth at a constant rate. The steady-state assumption, in turn, permits treatment of the ribosomal reaction complex (Fig. 1) as a cellular reaction subsystem, and to measure its output as an indicator of the subsystem function and therefore as a property of the subsystem. These two simplifications make it possible to treat ribosomal protein synthesis as a "black box" subsystem comprised by many component activities, and to use only the output of functional protein to measure this subsystem property. Any gene product can be used to measure a functional property of the protein synthesis subsystem. For example, the specific activity of a biosynthetic pathway enzyme can be used to measure the reaction property of specific protein output per unit of translating RNA associated with a single gene in a crude extract of a pure culture of a bacterium. If the *k*_cat _of the enzyme is known, i.e. reaction units per mg enzyme, the expression level, *λ*_*a*_, is the mass ratio of the amount of enzyme protein, measured by activity, to the total extract protein. The translation efficiency correlated to *ψ*_*a *_can be calibrated to a scale of codon usage relative to the highest expression set of genes. The definition of *ψ*_*a *_requires values for the codon usage measure to vary over the range 0 ≤ *ψ*_*a *_≤ 1.0, and several possible calculations of bias in codon usage previously reported could be used. As an example, the codon adaptation index of Sharp and Li [21] has been shown to correlate directly with the expression-level of protein-coding genes in *Escherichia coli *and may be used as a value for *ψ*_*a *_in this context [16]. We also suggest that a Pearson cross correlation measure of similarity between the relative codon frequency vector of a test gene, **a**, and a reference vector, **b**, of a high-expression gene set, i.e. *r*_*γ*_(**a**|**b**), may be well suited and sufficient for this measure.

Measurement of the activity of the functional protein output from a gene permits use of any protein-coding gene or set of genes to calibrate specific translation activity as the measurable reactive property or parameter of ribosomes, using *T*_*a*(max) _when *ρ*_*a *_is known or using ${\tilde{T}}_{a(\max)}$ when *ρ*_*a *_is unknown. It is reasonable to expect that measurement of ${\tilde{T}}_{a(\max)}$ as the specific translation activity for *m*_*a *_is fractionally related to *T*_*a*(max) _just as the specific activity of an enzyme measured in a crude cell extract, *V*_max_/*P*, is fractionally related to *k*_cat _= *V*_max_/*P*_*a *_for the purified enzyme. So, it is appropriate to treat the measurement of the specific translation activity, ${\tilde{T}}_{a(\max)}$, for bacterial ribosomes under defined growth conditions as one would treat measuring the specific activity, *V*_max_/*P*, for an enzyme under defined reaction conditions in a crude cell extract.

The ribosome reaction models for this study expect values for *T*_*a*(max) _and ${\tilde{T}}_{a(\max)}$ to vary according to the gene-specific mRNA undergoing translation and the specific reactive properties of the ribosomes within an organism. Owing to the *ρ*_*a *_relationship between *T*_*a*(max) _and ${\tilde{T}}_{a(\max)}$, what is true for ${\tilde{T}}_{a(\max)}$ is also true for *T*_*a*(max)_. Since it is likely that a value for *ρ*_*a *_will frequently be unknown, this discussion will treat ${\tilde{T}}_{a(\max)}$ as a proxy for *T*_*a*(max)_. A basic assumption of our model is that the reactive properties of ribosomes are constant within an organism under a defined set of growth conditions. The model pioneered by Kurland [20,18] and uniquely applied by Dethlefsen and Schmidt [11] is based upon treating the generalized protein production per unit time per total RNA (*kP*/*R*) as constant for a specific growth condition of an organism. If the apparent difference in translational power between ribosomes of a bacterium *A *compared to *B *can be attributed to differences in the ribosomes [11], we suggest that ${\tilde{T}}_{a(\max)}$ may improve the accuracy of measurement of the ribosomal differences and offer a way to study the natural variation in the properties associated with ribosomal reactivity. Our proposed improvement in accuracy is based upon the measurement of translational output, or power, from the mRNA from an individual gene in our ribosome reaction model. This model predicts that the natural variation in specific translation activity for mRNA from any genes within a genome should show a central tendency that is characteristic for that organism, such that

(29) $${\tilde{T}}_{a_{1}(\max)}\approx {\tilde{T}}_{a_{2}(\max)}\approx {\tilde{T}}_{a_{3}(\max)}\ldots \approx {\tilde{T}}_{a_{n}(\max)}$$

for specific genes *a*_1 _through *a*_*n *_in the genome of an organism *A*. This means, of course, that while the specific translation activity for different genes will be expected to vary within a genome, an average specific translation activity will represent the ribosome property for the host organism. According to Eq. (28), the steady state condition under which the relationships of Eq. (29) would hold requires that *λ*_*a*_/*ψ*_*a *_be approximately constant for all *a*_*i*_. This means, then, that if specific translation activity is an approximately constant property of the ribosomes of an organism, the ratio of the expression level to codon usage bias must also be approximately constant. This inference forms the basis of postulates that are experimentally testable and should reveal the natural variation in ${\tilde{T}}_{a(\max)}$ within an organism.

Measurement of ${\tilde{T}}_{a(\max)}$ should also provide the basis to compare and assess the natural variation in ribosome reactivity using the same gene or set of genes in all organisms. For that purpose, crude extracts of steady-state growth cultures will yield ${\tilde{T}}_{a(\max)}$ values for each defined growth condition to compare with ${\tilde{T}}_{a(\max)}$ for a different organism under a different growth condition. Expect to observe, measuring the same protein for *n *different bacterial genera, that $T_{a(\max)}^{\left(1\right)},T_{a(\max)}^{\left(2\right)},T_{a(\max)}^{\left(3\right)},\ldots T_{a(\max)}^{\left(\ldots \right)},T_{a(\max)}^{\left(n\right)}$ comprise a skewed frequency distribution that characterizes the natural variation in this ribosomal reactive property.

In addition to using the reaction rate measurement *T*_*a*(max) _and ${\tilde{T}}_{a(\max)}$ to characterize ribosomes, it should be possible to measure a *K*_m _for the ribosome reaction model by using in vitro translation with rate-limiting concentrations of an aminoacylated tRNA as conceptually depicted in Fig. 1. Measurement of a *K*_m _for a charged tRNA would then allow characterization of a ribosome reaction efficiency as *T*_*a*(max)_/*K*_m _or ${\tilde{T}}_{a(\max)}$/*K*_m_. A further advantage of this model is that it corrects for various specific errors, e.g. translational frameshift and other output errors, by basing the ribosome reactivity upon production of functional enzymes and measuring those functions directly.

A useful model should reflect the behavior of a system under naturally occurring as well as experimental conditions. One observation is that ribosomes are rarely saturated under a specific growth condition [11]. Achievement of a steady-state proposed by this model requires only a constant balance of reactants to achieve a stable output over time. This steady-state model applies for all degrees of saturation. This Ribosome Reaction model, $T_{a(\max)}=\frac{kP\lambda_{a}}{\rho_{a}R\psi_{a}}$, behaves such that high speed of translation is associated with high values for the growth rate, *k*, and expression level *λ*_*a*_, and low values for the translation efficiency *ψ*_*a*_. Conversely, slower translation speed is associated with slower growth, *k*, lower expression levels, *λ*_*a*_, and higher translation efficiency, *ψ*_*a*_. In other words, organisms that are rapid responders to change of environmental conditions will tend to have sloppier translation than slow responders [12], and our Ribosome Reaction model supports these translation relationships.

Development of this model provides a potential boost to successful inference of physiology from genomic sequence. If the relationships of Eq. (27) and the proportionate measurement of Eq. (28) hold in analysis of codon usage and gene expression level, it may become possible to associate specific ribosomal characteristics to bacterial lifestyle and ecological niche, i.e. to establish that ribosomes are customized for their host environment. For example, using a sufficient number of measurements of threonine deaminase specific activity in steady-state growth of a phylogenetically varied set of bacteria, along with codon usage patterns of these genes, the calculated value of *T*_*a*(max) _may distribute into expected ranges that are consistent with specific lifestyles from mostly dormant to rapid responders. This model provides an accessible tool and potential to measure a physiological bias of ribosomes to phylogeny and the environment in which they operate.

## Abbreviations

Except in Fig. 1 and noted in the Fig. 1 legend, only standard abbreviations were used in this paper.

## Authors' contributions

JHJ, TMS and PAH fully shared in all aspects of this study to develop a quantitative model of bacterial translation to measure natural variation in specific protein output by ribosomes. JHJ led in the model development, TMS contributed specific expertise in ribosomal properties, and PAH brought perspectives on enzyme reaction models into the study. All authors shared in all phases of evolution of the concepts, approaches, and the final construct of the model, as well as preparation of the manuscript.
